# Modeling and validation of wearable sensor-based gait parameters in Parkinson’s disease patients with cognitive impairment

**DOI:** 10.3389/fnagi.2025.1590224

**Published:** 2025-07-25

**Authors:** Guo Hong, Fengju Mao, Mingming Zhang, Fei Zhang, Xiangcheng Wang, Kang Ren, Zhonglue Chen, Xiaoguang Luo

**Affiliations:** ^1^Department of Neurology, Shenzhen People’s Hospital (The Second Clinical Medical College, Jinan University, The First Affiliated Hospital, Southern University of Science and Technology), Shenzhen, China; ^2^Shenzhen Clinical Research Centre for Geriatrics, Shenzhen People’s Hospital, Shenzhen, China; ^3^Shenzhen Key Laboratory of Smart Healthcare Engineering, Department of Biomedical Engineering, Southern University of Science and Technology, Nanshan, Shenzhen, Guangdong, China; ^4^Gyenno Science Co., Ltd., Shenzhen, Guangdong, China; ^5^HUST-GYENNO CNS Intelligent Digital Medicine Technology Center, School of Artificial Intelligence and Automation, Huazhong University of Science and Technology, Wuhan, Hubei, China; ^6^Department of Nuclear Medicine, Shenzhen People’s Hospital (The Second Clinical Medical College, Jinan University; The First Affiliated Hospital, Southern University of Science and Technology), Shenzhen, Guangdong, China

**Keywords:** Parkinson’s disease, cognitive impairment, gait parameters, wearable sensors, machine learning

## Abstract

**Background:**

Cognitive impairment is a common non-motor symptom of Parkinson’s disease (PD) that significantly impacts patients’ quality of life and disease progression. Despite its clinical importance, the underlying mechanisms linking motor and cognitive dysfunction in PD remain poorly understood. Wearable sensor technology offers an innovative approach to quantifying gait parameters and exploring their relationship with cognitive decline, providing a non-invasive, objective method to identify individuals at risk of cognitive impairment.

**Objective:**

This study aimed to develop and validate a diagnostic model using gait parameters derived from wearable sensors to predict cognitive impairment in PD patients. Additionally, it sought to integrate these findings with machine learning methods to enhance prediction accuracy.

**Methods:**

A cross-sectional study was conducted on early-to-mid-stage PD patients, with approximately 28.8% diagnosed with cognitive impairment. A total of 38 clinically relevant variables were collected, including demographic data, medical history, cognitive scale scores, and gait data captured by wearable sensors. Baseline comparisons, univariate, and multivariate logistic regression analyses were performed to identify independent risk factors for cognitive impairment. Selected variables were used to train and evaluate six machine-learning models. The models’ predictive performance was comprehensively assessed using receiver operating characteristic (ROC) curves, area under the curve (AUC) values, decision curve analysis (DCA), calibration curves, precision-recall (PR) curves, and forest plots. Shapley Additive Explanations (SHAP) analysis was also employed to enable personalized risk assessment. Finally, correlations between cognitive scores (MoCA and MMSE) and key gait parameters were analyzed.

**Results:**

Among the 38 clinical variables, seven were identified as independent risk factors for cognitive impairment in PD, including Duration of PD, UPDRS-III score, Step Length, Walk speed, Stride time, Peak arm angular velocity, Peak angular velocity during steering. The logistic regression model demonstrated superior predictive performance (test set AUC: 0.957), outperforming other machine learning algorithms. SHAP analysis revealed that Step Length, UPDRS-III score, Duration of PD, and Peak angular velocity during steering were the most influential predictors in the logistic regression model. Additionally, correlation analysis showed a significant association between lower cognitive scores and deteriorating gait parameters.

**Conclusion:**

This study highlights the potential of gait parameters derived from wearable sensors as biomarkers for cognitive impairment in PD patients. It also underscores the intricate interplay between motor and cognitive dysfunction in PD. The integration of gait analysis with machine learning models, particularly logistic regression, provides a robust, non-invasive, and scalable approach for early identification and risk stratification of cognitive decline in PD. By leveraging wearable technology, this work paves the way for innovative diagnostic strategies to enhance clinical decision-making and improve patient outcomes.

## 1 Introduction

Parkinson’s disease (PD) is a neurodegenerative disorder primarily affecting motor function, but cognitive impairment frequently co-occurs as the disease progresses (Booth et al., 2022). The relationship between motor dysfunction and cognitive deficits has garnered significant attention due to its profound impact on patient management and quality of life. Recent studies indicate that approximately one-quarter of early-stage PD patients experience cognitive impairment, underscoring the critical need for early identification and intervention strategies (Litvan et al., 2012; Martin et al., 2020). Wearable technology has emerged as a promising tool for assessing gait parameters and offers the potential for evaluating both motor and cognitive functions in PD patients. By capturing real-time data on movement patterns, these devices provide valuable insights into subtle changes that may signal the onset of cognitive decline. Previous studies have demonstrated that gait abnormalities in PD are not only associated with motor deficits but may also reflect underlying cognitive dysfunction (Bruni et al., 2022; Lunardini et al., 2021). As cognitive processing is integral to motor control, deviations in gait patterns may serve as markers of cognitive impairment. Although these findings suggest that gait disturbances could be sensitive indicators of early cognitive changes, the relationship between gait parameters and cognitive decline in PD remains underexplored. As PD progresses, these gait abnormalities often worsen, typically preceding overt cognitive impairment, thereby providing a window for early detection. Machine learning (ML) methods have been increasingly applied in various biomedical research and clinical practice fields, including neurology, to improve diagnostic accuracy (Hsiung et al., 2023). The integration of ML with wearable sensor data has opened new avenues for developing predictive models for cognitive impairment in PD patients. While the existing literature has explored various ML algorithms, few studies have focused on their application in predicting cognitive decline using gait analysis. In our study, we aimed to develop a diagnostic model for identifying cognitive impairment in PD patients by combining gait parameters captured by wearable sensors with machine learning methods.

## 2 Materials and methods

### 2.1 Research subjects

A total of 177 clinically diagnosed Parkinson’s disease (PD) patients who attended the Department of Neurology at Shenzhen People’s Hospital, Guangdong Province (Second Clinical Hospital of Jinan University and First Affiliated Hospital of Southern University of Science and Technology), between April 2023 and November 2024, were enrolled as the primary study cohort.

#### 2.1.1 Inclusion and exclusion criteria for PD patients

*Inclusion criteria*: (1) Diagnosis of PD confirmed by two movement disorder specialists based on the International Parkinson and Movement Disorder Society (MDS) criteria (Postuma et al., 2015); (2) Age ≥ 18 years; (3) Ability to walk independently; (4) Availability of complete and accurate clinical data; (5) Full capacity to provide autonomous informed consent.

*Exclusion criteria*: (1) History of neurological or psychiatric disorders that could interfere with the diagnosis or treatment of PD; (2) History of deep brain stimulation (DBS) or other invasive brain surgeries; (3) Any recent (< 4 weeks) initiation or dose adjustment of antiparkinsonian drugs; Use of anticholinergic medications for any indication; Use of cholinesterase inhibitors (e.g., donepezil, rivastigmine, galantamine) or NMDA antagonists (e.g., memantine); Any other central nervous system (CNS)-active medications known to influence gait or cognition, such as sedatives, antipsychotics, or antidepressants with anticholinergic properties; (4) Presence of severe systemic diseases (e.g., cardiac, hepatic, renal), symptomatic orthostatic hypotension, or other gait-impairing conditions; (5) History of substance abuse or alcoholism; (6) Other medical conditions potentially impacting study outcomes.

#### 2.1.2 Cognitive impairment classification in PD patients

Cognitive impairment was defined using the Mini-Mental Status Examination (MMSE) as a score below the education-adjusted threshold (Kochhann et al., 2010). Specifically, the cutoff scores were defined as follows: 21 for illiterate individuals, 22 for individuals with low education (1–5 years), 23 for those with moderate education (6–11 years), and 24 for those with high education (≥ 12 years). Based on these thresholds, PD patients were classified into two groups: those without cognitive impairment and those with cognitive impairment.

### 2.2 Collection of clinical baseline data

Clinical baseline data were collected for all enrolled participants, including (1) General characteristics: Sex, Smoking, Alcohol drinking, Age, Duration of PD), Unified Parkinson’s Disease Rating Scale Part III (UPDRS-III) score, and Levodopa-equivalent daily dose (LEDs); (2) Medical history: History of Hypertension, Atrial fibrillation (AF), Coronary heart disease (CHD), Diabetes, and Hyperlipidemia; (3) Cognitive assessment: Scores on the Mini-Mental Status Examination (MMSE) and the Montreal Cognitive Assessment (MoCA); (4) Gait data collection: All participants underwent gait analysis using wearable sensor-based devices. This study was approved by the ethics committee of the research institution (LL-KY-2023175-02).

### 2.3 Description, preparation, and usage of wearable sensor-based gait analysis equipment

**FIGURE 1:**
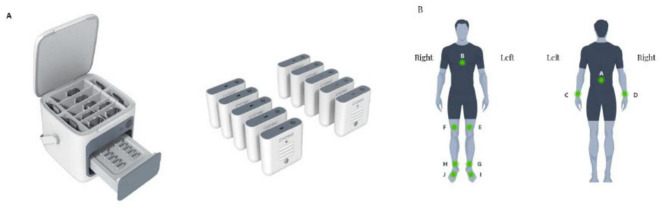
Illustration of a wearable sensor gait analysis device. **(A)** Sensor overview. **(B)** Sensor locations.

This research employed the MATRIX wearable motion and gait analysis system, developed by Gyenno Science in Shenzhen, China, which has obtained certifications from the National Medical Products Administration (NMPA), the U.S. Food and Drug Administration (FDA), and the European Conformité Européenne (CE) Medical standards (Gyenno Science Co., Ltd., 2022). The wearable gait sensor used in this study has been effectively verified in terms of accuracy and sensitivity in previous studies (Cai et al., 2023; Lin et al., 2023). During the experiment, each participant was equipped with ten inertial measurement unit (IMU) sensors operating at a sampling frequency of 100 Hz (as illustrated in Figure 1a). These IMU sensors collected inertial sensing data through a triaxial accelerometer (with a range of ± 16 g and a sensitivity of 16384 LSB/g) and a triaxial gyroscope (with a range of ± 2,000 dps and a sensitivity of 131 LSB/dps). The MATRIX system employs a centralized synchronization algorithm, wherein all IMU sensors are temporally aligned via a shared clock and real-time Bluetooth streaming, ensuring inter-sensor synchronization accuracy within ± 2 ms. Foot sensors were attached to the dorsum of each foot (metatarsal region). Thigh sensors were bilaterally positioned approximately 2 cm above the knees, while lower leg sensors were placed bilaterally about 2 cm above the ankle joints. Hand sensors were affixed to the dorsal side of each wrist. A chest sensor was secured on the sternum, and a lumbar sensor was positioned at the fifth lumbar vertebra. All sensors were fastened using adjustable straps (Figure 1b). Participants were instructed to complete a straight-line walking trial comprising an out-and-back course (16 m total distance, 8 m in one direction) at their self-selected comfortable speed under natural walking conditions. PD patients performed the test during their medication “on” phase. During the walking test, raw motion signals were captured in real-time via the 10 wearable sensors and transmitted to a central system for further analysis using a Bluetooth connection. Gait and posture transition parameters were automatically calculated from the raw motion signals using pre-established algorithms (Gyenno Science Co., Ltd., 2022).

All dependent gait variables were automatically extracted using validated proprietary algorithms embedded in the MATRIX software, which processed raw IMU signals in real-time to compute spatiotemporal, angular, symmetry, coordination, and steering-related parameters. The study primarily collected 24 gait parameters, including step length, walk speed, stride length, stride time, cadence, double support phase, swing phase, stance phase, peak shank angular velocity, shank symmetry index, relative mean phase difference, phase coordination index, inter-limb coordination, peak trunk coronal angular velocity, peak trunk sagittal angular velocity, peak trunk transverse angular velocity, peak trunk sway angle, peak arm angular velocity, arm symmetry index, average steering duration, average number of steps during steering, peak angular velocity during steering, average angular velocity during steering, and average time per step during steering.

### 2.4 Statistical methods

Statistical analyses were conducted using SPSS (version 27.0), R (version 4.2.3), and Python (version 3.11.4). Continuous variables with a normal distribution were expressed as mean ± standard deviation (× ± s) and compared using the *t*-test. Non-normally distributed continuous variables were expressed as the median and interquartile range (IQR) and compared using the Mann-Whitney U test. Categorical variables were presented as frequencies and percentages and analyzed using the chi-square test. PD patients were randomly divided into training and testing sets in a 7:3 ratio using the Pyskthon (0.22.1) random sampling method. To evaluate the multicollinearity among the independent variables, Pearson correlation analysis and variance inflation factor (VIF) assessment were conducted before multivariate logistic regression modeling. The VIF < 10 and r < 0.8 of the vast majority of variables are considered acceptable. A full correlation heatmap of the included variables is provided in Supplementary Figure 1 for transparency (The analysis results of Supplementary Figure 1 were generated using the CNSknowall platform,^1^ a comprehensive web service for data analysis and visualization). Univariate and multivariate logistic regression analyses were performed to identify independent risk factors for cognitive impairment in PD patients. The selected variables were incorporated into six machine-learning models for comparison. The predictive performance of these models was comprehensively evaluated using receiver operating characteristic (ROC) curves, area under the curve (AUC) values, decision curve analysis (DCA) curves, calibration curves, precision-recall (PR) curves, and forest plots. The optimal model was identified and interpreted using the SHAP (Shapley Additive exPlanations) interpreter. Finally, the correlations between MOCA and MMSE scores and independent risk factors were further analyzed (Correlation analyses were conducted across the full cohort to capture the continuous associations between motor parameters and cognitive function). All tests were two-tailed, with a significance threshold set at α = 0.05. A *P* < 0.05 was considered statistically significant.

## 3 Results

### 3.1 Comparison of baseline data between training and testing sets in PD patients

A total of 177 Parkinson’s disease (PD) patients were included in this study. Using the Pyskthon (0.22.1) random sampling method, the patients were randomly assigned to the training set (*n* = 123) and testing set (*n* = 54) in a 7:3 ratio. The detailed baseline characteristics of the training and testing sets are presented in Table 1. No significant differences were observed between the two groups (*P* > 0.05).

**TABLE 1:** Baseline characteristics in the training cohort and testing cohort.

Variables	Category item	Total (*n* = 177)	Training set (*n* = 123)	Testing set (*n* = 54)	Z	*P*
Cognitive impairment, *n*(%)	No	126(71.19)	87(70.73)	39(72.22)	0.041	0.840
	Yes	51(28.81)	36(29.27)	15(27.78)		
Sex, *n*(%)	Male	127(71.75)	88(71.55)	39(72.22)	0.008	0.927
	Female	50(28.25)	35(28.46)	15(27.78)		
Smoking, *n*(%)	No	135(76.27)	93(75.61)	42(77.78)	0.097	0.755
	Yes	42(23.73)	30(24.39)	12(22.22)		
Alcohol drinking, *n*(%)	No	130(73.45)	92(74.80)	38(70.37)	0.377	0.539
	Yes	47(26.55)	31(25.20)	16(29.63)		
Hypertension, *n*(%)	No	106(59.89)	76(61.79)	30(55.56)	0.607	0.436
	Yes	71(40.11)	47(38.21)	24(44.44)		
Diabetes, *n*(%)	No	162(91.53)	116(94.31)	46(85.19)	4.027	0.045
	Yes	15(8.48)	7(5.69)	8(14.82)		
Hyperlipidemia, *n*(%)	No	122(68.93)	89(72.36)	33(61.11)	2.216	0.137
	Yes	55(31.07)	34(27.64)	21(38.89)		
AF, *n*(%)	No	164(92.66)	112(91.06)	52(96.30)	1.514	0.219
	Yes	13(7.35)	11(8.94)	2(3.70)		
CHD, *n*(%)	No	163(92.09)	112(91.06)	51(94.44)	0.591	0.442
	Yes	14(7.91)	11(8.94)	3(5.56)		
Age (years) Median (IQR)		63.00 [57.00, 67.00]	64.00 [58.00, 68.00]	60.00 [53.00, 67.00]	1.843	0.065
Duration of PD (years) Median (IQR)		3.00 [3.00, 5.00]	3.00 [2.00, 5.00]	3.00 [3.00, 5.00]	−0.811	0.409
UPDRS-III (points) Median (IQR)		27.00 [22.00, 37.00]	27.00 [22.00, 38.00]	29.00 [22.00, 37.00]	0.113	0.911
LEDs (mg) Median (IQR)		435.00 [400.00, 510.00]	440.00 [400.00, 510.00]	425.00 [405.00, 515.00]	0.624	0.533
MOCA (points) Median (IQR)		27.00 [23.00, 28.00]	27.00 [23.00, 28.00]	26.00 [24.00, 28.00]	0.115	0.909
MMSE (points) Median (IQR)		25.00 [23.00, 27.00]	25.00 [23.00, 27.00]	25.00 [23.00, 28.00]	−0.358	0.720
Step length (cm) Median (IQR)		50.70 [41.61, 56.71]	50.70 [42.68, 55.67]	50.59 [37.40, 58.72]	0.021	0.985
Walk speed (m/s) Median (IQR)		0.84 [0.73, 1.06]	0.85 [0.74, 1.06]	0.83 [0.72, 1.06]	0.167	0.868
Stride length (cm) Median (IQR)		101.29 [85.36, 114.74]	100.99 [85.36, 114.74]	101.29 [77.23, 117.42]	−0.011	0.992
Stride time (s) Median (IQR)		1.13 [1.05, 1.20]	1.13 [1.05, 1.20]	1.12 [1.08, 1.20]	−0.591	0.556
Cadence (steps/min) mean ± SD		105.92 ± 10.15	106.04 ± 10.30	105.63 ± 9.80	0.245	0.807
Double support (%) Median (IQR)		20.26 [17.76, 22.55]	20.27 [17.77, 22.41]	19.55 [17.72, 22.71]	0.116	0.909
Swing (%) Median (IQR)		40.65 [39.38, 41.99]	40.59 [39.43, 42.09]	40.72 [39.25, 41.95]	−0.253	0.801
Stance (%) Median (IQR)		59.35 [58.01, 60.63]	59.41 [57.91, 60.57]	59.12 [58.05, 60.75]	0.253	0.801
Peak shank angular velocity (degree/s) Median (IQR)		318.82 [278.80, 349.96]	312.46 [279.79, 349.59]	321.47 [269.47, 350.03]	−0.387	0.700
Shank symmetry index (%) Median (IQR)		10.58 [8.98, 11.97]	10.68 [8.92, 11.87]	10.07 [9.04, 12.13]	0.457	0.649
Relative mean phase difference (%) Median (IQR)		3.63 [2.74, 5.05]	3.62 [2.74, 4.85]	3.63 [2.73, 5.16]	−0.393	0.695
Phase coordination index (%) Median (IQR)		5.56 [3.82, 7.30]	5.56 [3.80, 7.32]	5.27 [3.85, 7.25]	−0.255	0.800
Interlimb coordination (%) Median (IQR)		−2.32 [−5.72, 0.71]	−2.34 [−5.81, 0.70]	−2.11 [−4.95, 0.72]	−0.750	0.454
Peak trunk coronal angular velocity (degree/s) Median (IQR)		22.22 [18.48, 26.86]	22.61 [18.41, 27.10]	21.59 [19.25, 26.86]	−0.234	0.816
Peak trunk sagittal angular velocity(degree/s) Median (IQR)		32.59 [27.00, 39.57]	32.94 [27.64, 39.58]	31.42 [25.95, 39.36]	0.744	0.458
Peak trunk transverse angular velocity (degree/s) Mean ± SD		39.94 ± 7.91	40.17 ± 8.14	39.40 ± 7.31	0.593	0.554
Peak trunk sway angle (degree) Median (IQR)		4.39 [4.11, 5.02]	4.58 [4.11, 5.01]	4.32 [4.10, 5.20]	−0.032	0.976
Peak arm angular velocity (degree/s) median (IQR)		131.94 [111.16, 150.59]	132.06 [110.91, 150.85]	130.83 [121.16, 150.59]	−0.011	0.992
Arm symmetry index (%) Median (IQR)		39.04 [35.86, 41.67]	38.22 [34.49, 41.69]	39.48 [36.20, 41.14]	−0.742	0.459
Average steering duration (s) Median (IQR)		2.98 [2.59, 3.55]	2.98 [2.63, 3.54]	2.94 [2.56, 3.55]	0.147	0.885
Average number of steps during steering (steps) Median (IQR)		4.50 [4.00, 5.31]	4.50 [4.00, 5.50]	4.50 [4.00, 5.31]	−0.357	0.722
Peak angular velocity during steering (degree/s) Median (IQR)		102.17 [90.01, 124.73]	101.04 [90.00, 124.80]	102.17 [90.01, 124.47]	−0.454	0.651
Average angular velocity during steering (degree/s) Median (IQR)		60.69 [53.13, 71.56]	60.69 [53.13, 70.72]	59.49 [53.13, 73.10]	−0.210	0.835
Average time per step during steering (s) Median (IQR)		0.56 [0.51, 0.62]	0.56 [0.51, 0.63]	0.55 [0.51, 0.61]	0.725	0.470

AF, Atrial fibrillation; CHD, Coronary heart disease; PD, Parkinson’s disease; UPDRS-III, Unified Parkinson’s Disease Rating Scale Part III; LEDs, Levodopa Equivalent Daily Dose; MOCA, Montreal Cognitive Assessment Scale; MMSE, Mini-Mental Status Examination Scale. Data are presented as mean ± standard deviation (SD) for normally distributed variables, median (interquartile range) for non-normally distributed variables, and number (percentage) for categorical variables. Normality was tested using the Shapiro–Wilk test. Group comparisons were conducted using independent samples *t*-test, Mann–Whitney U test, or chi-square test, as appropriate.

### 3.2 Comparison of baseline data between PD patients with and without cognitive impairment

Using cognitive impairment as the grouping variable, a total of 177 Parkinson’s disease (PD) patients were included in the study. Among them, 126 patients were assigned to the normal cognitive group (NC), and 51 patients were assigned to the cognitive impairment group (CI). The CI group exhibited significantly higher values for age, duration of PD, UPDRS-III scores, LEDs, stride time, stance phase, peak trunk sway angle, average steering duration, and average number of steps during steering compared to the NC group (*P* < 0.05). In contrast, the NC group demonstrated significantly higher scores for MOCA, MMSE, step length, walk speed, stride length, swing phase, peak shank angular velocity, peak trunk sagittal angular velocity, peak arm angular velocity, arm symmetry index, peak angular velocity during steering, and average angular velocity during steering compared to the CI group (*P* < 0.05). Detailed data are presented in Table 2.

**TABLE 2:** Comparison of baseline data between groups of PD patients with and without cognitive impairment.

Variables	Category item	NC (*n* = 126)	CI (*n* = 51)	Z	*P*
Sex, *n* (%)	Male	93 (73.81)	34 (66.67)	0.914	0.339
	Female	33 (26.19)	17 (33.33)		
Smoking, *n* (%)	No	92 (73.02)	43 (84.31)	2.560	0.110
	Yes	34 (26.98)	8 (15.69)		
Alcohol drinking, *n* (%)	No	91 (72.22)	39 (76.47)	0.336	0.562
	Yes	35 (27.78)	12 (23.53)		
Hypertension, *n* (%)	No	77 (61.11)	29 (56.86)	0.273	0.601
	Yes	49 (38.89)	22 (43.14)		
Diabetes, *n* (%)	No	116 (92.06)	46 (90.20)	0.163	0.686
	Yes	10 (7.94)	5 (9.80)		
Hyperlipidemia, *n* (%)	No	87 (69.05)	35 (68.63)	0.003	0.956
	Yes	39 (30.95)	16 (31.37)		
AF, *n* (%)	No	118 (93.65)	46 (90.20)	0.637	0.425
	Yes	8 (6.35)	5 (9.80)		
CHD, *n* (%)	No	116 (92.06)	47 (92.16)	0.000	0.983
	Yes	10 (7.94)	4 (7.84)		
Age (years) Median (IQR)		62.00 [54.00, 67.00]	65.00 [60.00, 68.00]	−2.400	0.016
Duration of PD (years) Median (IQR)		3.00[2.00, 4.00]	5.00[4.00, 6.00]	−5.991	< 0.001
UPDRS-III (points) Median (IQR)		24.00 [19.00, 29.00]	38.00 [36.00, 44.00]	−8.418	< 0.001
LEDs (mg) Median (IQR)		415.00 [390.00, 440.00]	525.00 [520.00, 540.00]	−10.405	< 0.001
MOCA (points) Median (IQR)		27.00 [26.00, 28.00]	21.00 [19.00, 22.00]	10.407	< 0.001
MMSE (points) Median (IQR)		27.00 [25.00, 28.00]	21.00 [20.00, 22.00]	10.407	< 0.001
Step length (cm) Median (IQR)		52.29 [49.42, 58.83]	35.84 [28.76, 44.26]	8.480	< 0.001
Walk speed (m/s) Median (IQR)		0.93[0.82, 1.08]	0.68[0.48, 0.74]	8.642	< 0.001
Stride length (cm) Median (IQR)		106.43 [100.93, 117.90]	72.68 [55.06, 89.34]	8.541	< 0.001
Stride time (s) Median (IQR)		1.10[1.04, 1.19]	1.15[1.09, 1.26]	−2.847	0.004
Cadence (steps/min) Median (IQR)		108.51 [101.11, 112.81]	106.20 [97.58, 113.84]	1.402	0.161
Double support (%) Median (IQR)		19.46 [17.67, 22.46]	20.67 [17.77, 22.70]	−1.545	0.123
Swing (%) Median (IQR)		40.82 [39.49, 42.14]	40.35 [38.72, 41.90]	2.222	0.026
Stance (%) Median (IQR)		59.18 [57.87, 60.51]	59.60 [58.10, 61.28]	−2.222	0.026
Peak shank angular velocity (degree/s) Median (IQR)		321.90 [296.91, 350.84]	278.14 [230.71, 328.15]	4.318	< 0.001
Shank symmetry index (%) Median (IQR)		10.68 [9.14, 12.11]	9.27 [6.97, 11.82]	1.862	0.063
Relative mean phase difference (%) Median (IQR)		3.648 [3.093, 4.741]	3.087 [2.494, 5.442]	0.551	0.583
Phase coordination index (%) Median (IQR)		5.56 [4.07, 7.21]	5.17 [3.11, 7.66]	0.424	0.673
Interlimb coordination (%) Median (IQR)		−2.32 [−5.73, 1.31]	−2.34 [−5.72, −0.69]	0.819	0.413
Peak trunk coronal angular velocity (degree/s) Median (IQR)		22.61 [19.42, 27.26]	21.37 [18.41, 24.64]	1.104	0.270
Peak trunk sagittal angular velocity (degree/s) Median (IQR)		34.51 [29.20, 40.74]	29.09 [25.95, 34.50]	3.417	< 0.001
Peak trunk transverse angular velocity (degree/s) Median (IQR)		40.46 [34.79, 46.03]	36.77 [34.96, 43.36]	1.360	0.174
Peak trunk sway angle (degree) Median (IQR)		4.31 [4.09, 4.98]	4.92 [4.31, 5.53]	−3.281	0.001
Peak arm angular velocity (degree/s) Median (IQR)		139.00 [123.83, 151.79]	121.04 [94.47, 136.85]	3.378	< 0.001
Arm symmetry index (%) Mean ± SD		39.31 ± 4.80	37.64 ± 3.62	2.226	0.027
Average steering duration (s) Median (IQR)		2.92[2.57, 3.38]	3.46[2.81, 5.60]	−3.414	< 0.001
Average number of steps during steering (steps) Median (IQR)		4.40[4.00, 5.15]	5.00[4.00, 7.00]	−2.340	0.019
Peak angular velocity during steering (degree/s) Median (IQR)		106.37 [91.14, 125.25]	95.38 [79.67, 117.59]	2.930	0.003
Average angular velocity during steering (degree/s) Mean ± SD		63.22 ± 10.31	52.63 ± 17.90	3.932	< 0.001
Average time per step during steering (s) Median (IQR)		0.55[0.51, 0.58]	0.58[0.50, 0.67]	−0.904	0.367

AF, Atrial fibrillation; CHD, Coronary heart disease; PD, Parkinson’s disease; UPDRS-III, Unified Parkinson’s Disease Rating Scale Part III; LEDs, Levodopa Equivalent Daily Dose; MOCA, Montreal Cognitive Assessment Scale; MMSE, Mini-Mental Status Examination Scale.

### 3.3 Univariate and multivariate logistic regression analysis

Cognitive impairment in PD patients was used as the dependent variable, while independent variables included age, duration of PD, UPDRS-III scores, LEDs, MOCA, MMSE, step length, walk speed, stride length, stride time, swing phase, stance phase, peak shank angular velocity, peak trunk sagittal angular velocity, peak trunk sway angle, peak arm angular velocity, arm symmetry index, average steering duration, average number of steps during steering, peak angular velocity during steering, and average angular velocity during steering. Univariate logistic regression analysis revealed significant associations between cognitive impairment and age, duration of PD, UPDRS-III, step length, walk speed, stride length, stride time, swing phase, stance phase, peak shank angular velocity, peak trunk sagittal angular velocity, peak arm angular velocity, arm symmetry index, average steering duration, average number of steps during steering, peak angular velocity during steering, and average angular velocity during steering (*P* < 0.05). After adjusting for other variables, multivariate logistic regression analysis demonstrated that duration of PD, UPDRS-III, step length, walk speed, stride time, peak arm angular velocity, and peak angular velocity during steering remained significantly associated with cognitive impairment in PD patients (*P* < 0.05). These indicators may serve as independent risk factors for predicting cognitive impairment in PD patients (Table 3).

**TABLE 3:** Univariate and multivariate logistic regression analysis.

Variables	Univariate analysis			Multivariate analysis		
	OR	95%CI	*P*	OR	95%CI	*P*
Age	1.051	1.009, 1.095	0.016			
Duration of PD	1.781	1.422, 2.230	0.000	1.953	1.364, 3.020	0.001
UPDRSIII	1.236	1.159, 1.317	0.000	1.320	1.178, 1.535	< 0.001
LEDs	74.318	0.000, inf	0.999			
MOCA	0.000	0.000, inf	0.999			
MMSE	0.000	0.000, inf	0.999			
Step length	0.826	0.781, 0.873	0.000	0.684	0.539, 0.852	0.001
Walk speed	0.000	0.000, 0.001	0.000	2.671 × 10^6^	3.698, 3.686 × 10^12^	0.030
Stride length	0.911	0.887, 0.937	0.000			
Stride time	157.813	7.471, 3333.678	0.001	5.115 × 10^8^	2.471 × 10^4^, 8.712 × 10^13^	< 0.001
Swing	0.770	0.645, 0.920	0.004			
Stance	1.299	1.087, 1.551	0.004			
Peak shank angular velocity	0.983	0.975, 0.990	0.000			
Peak trunk sagittal angular velocity	0.933	0.895, 0.973	0.001			
Peak trunk sway angle	1.285	0.948, 1.741	0.106			
Peak arm angular velocity	0.982	0.972, 0.993	0.001	0.979	0.958, 0.995	0.029
Arm symmetry index	0.919	0.851, 0.992	0.029			
Average steering duration	2.216	1.536, 3.198	0.000			
Average number of steps during steering	1.630	1.281, 2.073	0.000			
Peak angular velocity during steering	0.976	0.960,0.992	0.004	1.055	1.011, 1.110	0.023
Average angular velocity during steering	0.943	0.919, 0.969	0.000			

OR, Odds ratio; CI, Confidence interval; PD, Parkinson’s disease; UPDRS-III, Unified Parkinson’s Disease Rating Scale Part III; LEDs, Levodopa Equivalent Daily Dose; MOCA, Montreal Cognitive Assessment Scale; MMSE, Mini-Mental Status Examination Scale.

### 3.4 Comprehensive analysis of classification models

**FIGURE 2:**
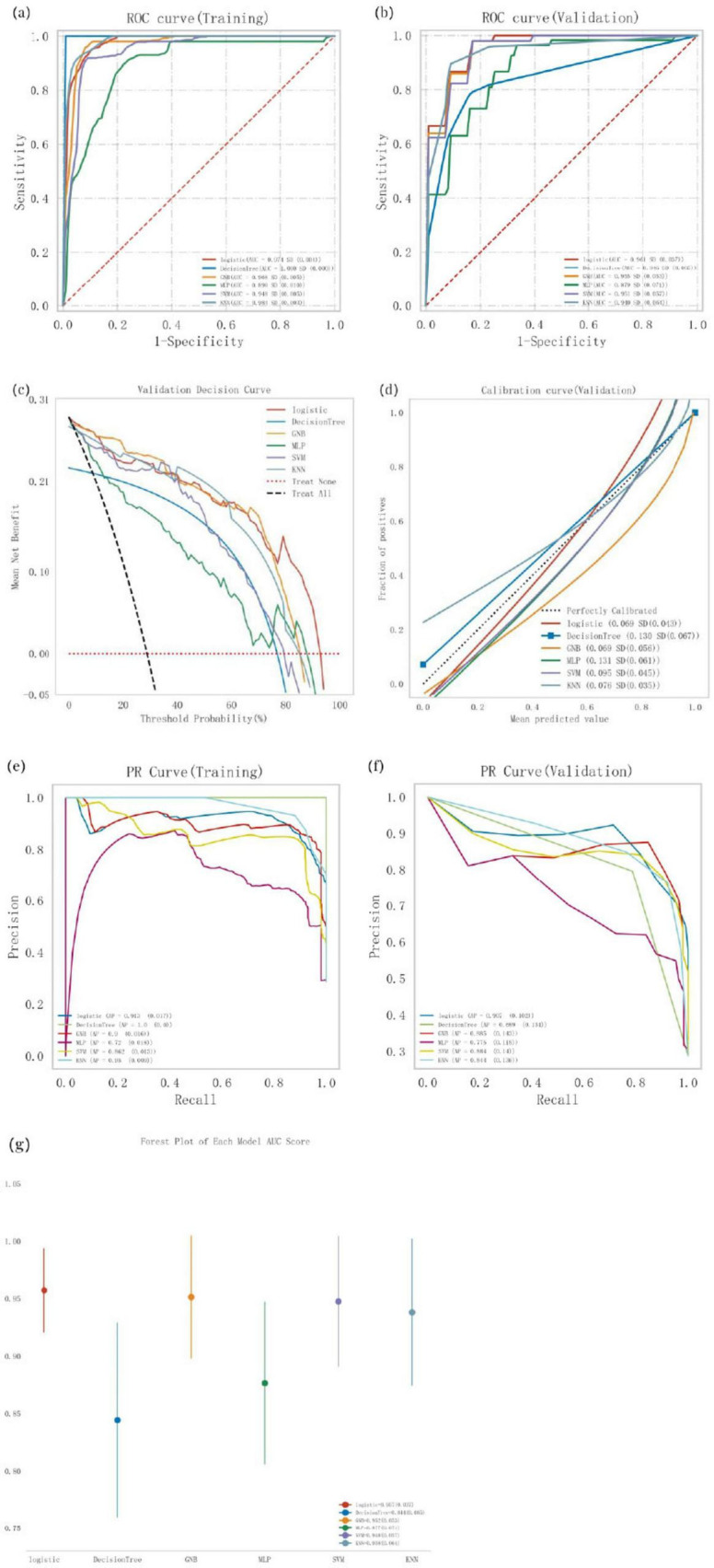
Comprehensive analysis of classification models, **(a)** Receiver operating characteristic (ROC) curves and area under the curve (AUC) for the training set; **(b)** ROC curves and AUC for the validation set. PD patients were sampled 10 tunes using a 7:3 split ratio, **(c)** Decision curve analysis (DCA) for the validation set, where the black dashed line assumes that all patients have cognitive impairment, and the red dashed line assumes no patients have cognitive impairment. Die remaining solid lines represent different models, **(d**) Calibration curves for the validation set, where the *x*-axis represents the mean predicted probability, and the *y*-axis represents the actual probability of the event. The dashed diagonal line serves as the reference line, while the remaining smoothed solid lines represent the fitted lines for different models. A fitted line closer to the reference line, with smaller values in parentheses, indicates greater predictive accuracy of the model, **(e)** Precision-recall (PR) curve and average precision (AP) for the training set; **(f)** PR curve and AP for the validation set. The y-axis denotes precision, while the x-axis represents recall. If the PR curve of one model is completely covered by another, the latter is superior. Higher AP values indicate better model performance. Different colors in the figure represent different models, **(g)** Forest plot comparing the AUC scores and confidence intervals of various models.

Logistic regression, Decision Tree, Gaussian naive Bayes (GNB), multi-layer perceptron (MLP), support vector machine (SVM), and k-nearest neighbor (KNN) models were trained and evaluated through 10 repeated iterations. The models were assessed using the area under the curve (AUC) metric, which demonstrated that Decision Tree, KNN, and Logistic regression achieved the highest AUC values in the training dataset, while Logistic regression achieved the highest AUC in the validation dataset (Table 4; Figures 2a,b). While AUC emphasizes predictive accuracy, it does not provide insight into the model’s clinical applicability or which model is preferable. Therefore, additional analyses including decision curve analysis (DCA), calibration curves, precision-recall (PR) curves, and forest plots were performed. DCA indicated superior clinical utility for Logistic regression and GNB (Figure 2c). Calibration curves also confirmed higher predictive accuracy for Logistic regression and GNB (Figure 2d). Across both training and validation datasets, the Logistic regression model consistently showed strong performance, achieving the highest average precision (AP) value in the validation dataset (Figures 2e,f). The forest plot revealed notable differences in AUC scores among models, with Logistic regression, GNB, SVM, and KNN achieving high AUC values, all exceeding 0.9 (Figure 2g). Comprehensive analysis suggests that Logistic regression can be considered the optimal model.

**TABLE 4:** Classification results of multiple models (including training set and validation set).

Classification model	AUC (SD)	Cutoff (SD)	Accuracy (SD)	Sensitivity (SD)	Specificity (SD)
**Training set**					
Logistic	0.974 (0.004)	0.241 (0.039)	0.911 (0.013)	0.943 (0.028)	0.898 (0.024)
Decision tree	1.000 (0.000)	1.000 (0.000)	0.712 (0.002)	0.000 (0.000)	1.000 (0.000)
GNB	0.968 (0.005)	0.256 (0.081)	0.928 (0.011)	0.950 (0.010)	0.920 (0.017)
MLP	0.890 (0.010)	0.186 (0.047)	0.817 (0.013)	0.885 (0.039)	0.790 (0.026)
SVM	0.948 (0.005)	0.393 (0.032)	0.920 (0.009)	0.893 (0.018)	0.931 (0.010)
KNN	0.984 (0.003)	0.580 (0.140)	0.921 (0.042)	0.803 (0.184)	0.968 (0.025)
**Validation set**					
Logistic	0.961 (0.037)	0.241 (0.039)	0.881 (0.081)	0.927 (0.117)	0.864 (0.100)
Decision tree	0.846 (0.085)	1.000 (0.000)	0.712 (0.017)	0.000 (0.000)	1.000 (0.000)
GNB	0.955 (0.053)	0.256 (0.081)	0.909 (0.064)	0.940 (0.092)	0.896 (0.081)
MLP	0.879 (0.071)	0.186 (0.047)	0.786 (0.089)	0.827 (0.158)	0.770 (0.075)
SVM	0.951 (0.057)	0.393 (0.032)	0.897 (0.072)	0.850 (0.175)	0.920 (0.072)
KNN	0.940 (0.064)	0.580 (0.140)	0.876 (0.069)	0.733 (0.263)	0.937 (0.067)

### 3.5 Establishment and evaluation of the optimal model

**FIGURE 3:**
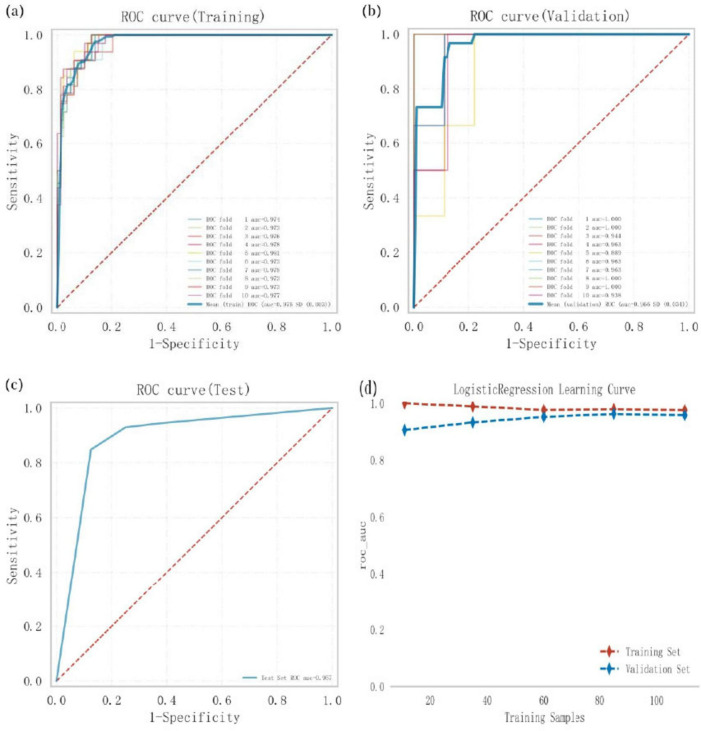
Logistic regression model training. Validation, and testing, **(a)** Receiver operating characteristic (ROC) curve and area under the curve (AUC) for the training set. **(b)** ROC and AUC for the validation set, where solid lines of different colors represent 10 distinct results, **(c)** ROC and AUC for the test set, based on results from 30% of PD patients, **(d)** Learning curves, with the red dashed line representing the training set and the blue dashed line representing the validation set.

Logistic regression analysis combined with 10-fold cross-validation was conducted on the training set. The results showed an average area under the curve (AUC) of 0.975 for the training set, 0.966 for the validation set, and 0.957 for the test set (Figures 3a–c). The AUC values for the training, validation, and test sets stabilized around 0.96, indicating the high predictive accuracy of the model. Furthermore, when the AUC performance of the validation set is less than 10% lower than that of the test set, the model can be considered successfully fitted. The learning curves demonstrated strong fitting ability and high stability for both the training and validation sets (Figure 3d). These findings suggest that the logistic regression model outperforms other models in terms of predictive accuracy and can be effectively applied to classification tasks for this dataset.

### 3.6 SHAP model interpretation

**FIGURE 4:**
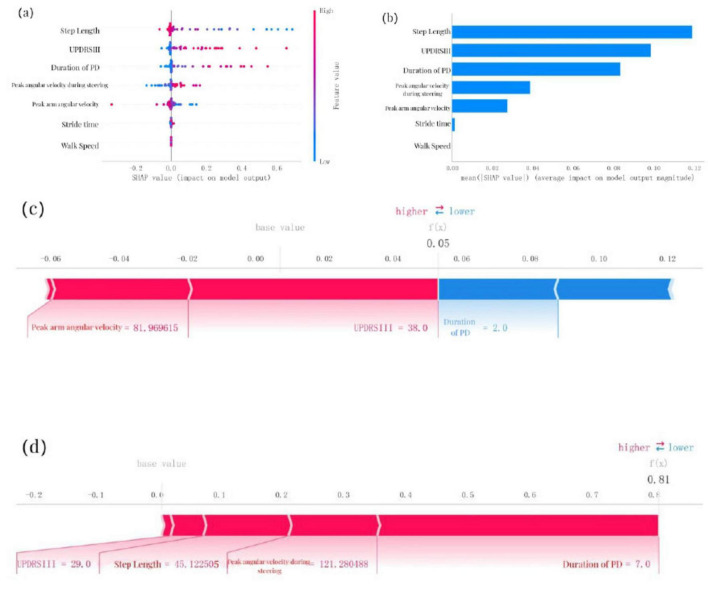
SHAP model interpretation, **(a)** Feature attributions in SHAP. Each line represents a feature, with the *x*-axis indicating SHAP values. Red dots correspond to high feature values, while blue dots indicate low feature values, **(b)** Feature importance ranking represented by SHAP. The matrix plot illustrates the significance of each covariate in developing the final predictive model, **(c)** Interpretability model for a PD patient without cognitive impairment and **(d)** a PD patient with cognitive impairment. The numbers below f(x) represent the predicted probability values, while the baseline value reflects the prediction without any input to the model. Red features indicate increased risk, whereas blue features signify reduced risk. The length of the arrows provides a visual representation of the degree to which predictions are influenced; longer arrows indicate greater impact.

To provide an intuitive explanation of the selected variables, SHAP (Shapley Additive exPlanations) was used to illustrate how these variables predict the presence of cognitive impairment within the model. Figure 4a highlights the top seven features in our model. Each feature importance line represents all patients’ attributions to the outcome, with red dots indicating high-risk values and blue dots indicating low-risk values. Decreases in step length, increases in UPDRS-III scores, longer duration of PD, higher peak angular velocity during steering, reductions in peak arm angular velocity, increases in stride time, and decreases in walk speed were associated with a higher likelihood of cognitive impairment in PD patients. Figure 4b ranks the seven risk factors based on their mean absolute SHAP values, with the x-axis SHAP values indicating their importance to the predictive model. Additionally, two representative cases are presented to illustrate the model’s interpretability. One example depicts a PD patient without cognitive impairment, showing low predictive efficiency [f(x) = 0.05] (Figure 4c). The other example illustrates a PD patient with cognitive impairment, demonstrating high predictive efficiency [f(x) = 0.81] (Figure 4d).

### 3.7 Correlation analysis between cognitive levels and motor parameters

**FIGURE 5:**
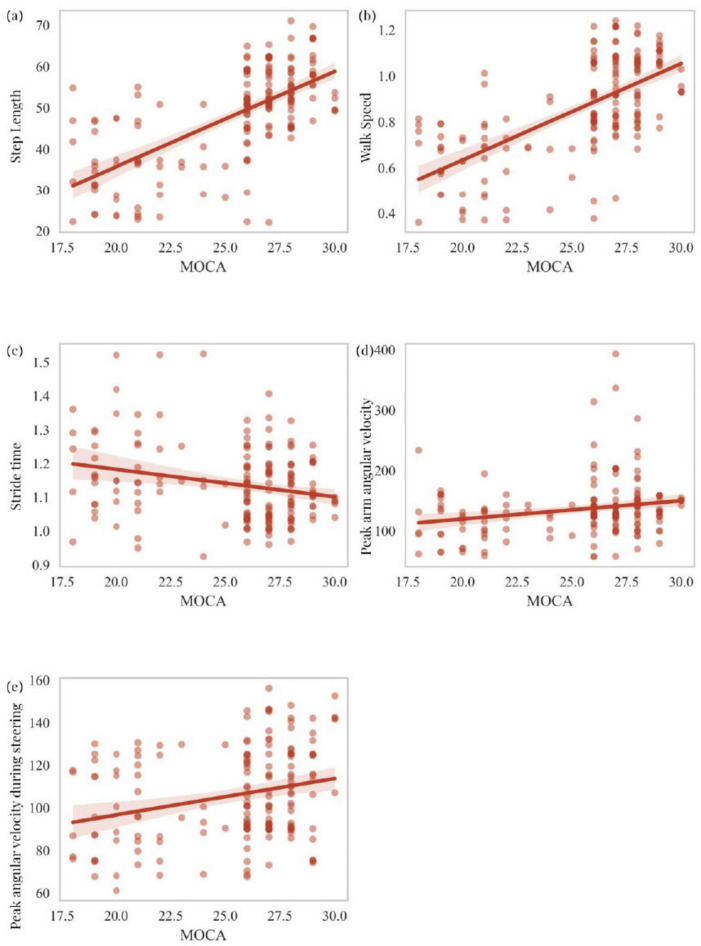
Correlation analysis of MOCA scores with step length, walk speed, stride time, peak arm angular velocity, and peak angular velocity during steering **(a–e)**.

**FIGURE 6:**
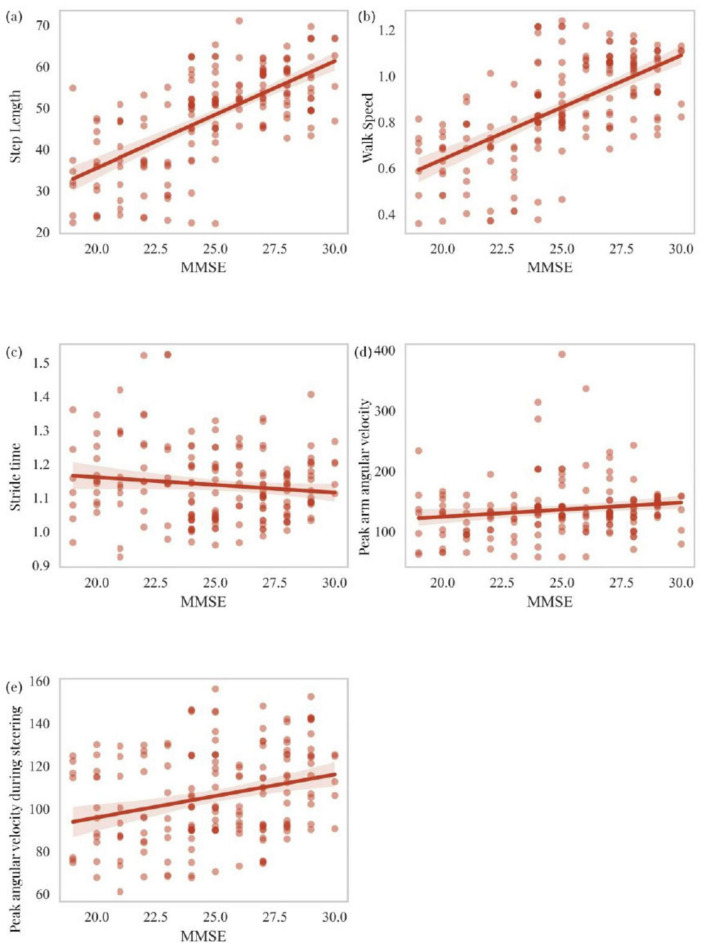
Correlation analysis of MMSE scores with step length, walk speed, stride time, peak arm angular velocity, and peak angular velocity during steering **(a–e)**.

This section primarily analyzes the correlation between scores on two cognitive scales, MOCA and MMSE, and five motor-related independent risk factors identified through logistic regression analysis as predictors of cognitive impairment in PD patients. These factors include step length, walk speed, stride time, peak arm angular velocity, and peak angular velocity during steering. MOCA scores showed a statistically significant positive correlation with step length (*R* = 0.461, *P* < 0.001), walk speed (*R* = 0.640, *P* < 0.001), and peak arm angular velocity (*R* = 0.229, *P* = 0.002), while a significant negative correlation was observed with stride time (*R* = -0.174, *P* = 0.020). MOCA scores were also positively correlated with peak angular velocity during steering (*R* = 0.264, *P* < 0.001) (Figures 5a–e). Similarly, MMSE scores exhibited a statistically significant positive correlation with step length (*R* = 0.643, *P* < 0.001), walk speed (*R* = 0.641, *P* < 0.001), and peak arm angular velocity (*R* = 0.195, *P* = 0.009). However, no significant correlation was found between MMSE scores and stride time (*R* = -0.078, *P* = 0.301). MMSE scores also showed a statistically significant positive correlation with peak angular velocity during steering (*R* = 0.296, *P* < 0.001) (Figures 6a–e). To explain the potential anchoring effect of MMSE-based grouping, we conducted a correlation analysis for subgroups. Interestingly, many of these analyses lack statistical significance, which may be attributed to the reduction in sample size and the limitation of score variability within subgroups, thereby limiting the power of detecting correlation (see Supplementary material).

## 4 Discussion

This study developed diagnostic models for Parkinson’s disease (PD) patients with cognitive impairment by integrating gait parameters derived from wearable sensors with multiple machine-learning methods. Our results demonstrated that, through baseline comparison, univariate, and multivariate logistic regression analyses, seven clinical variables were identified from 38 clinical features, including duration of PD, UPDRS-III, step length, walk speed, stride time, peak arm angular velocity, and peak angular velocity during steering. These variables were determined to be independent risk factors for predicting cognitive impairment in PD patients, enabling risk assessment for cognitive dysfunction. Our study primarily focused on early- to mid-stage PD patients, with approximately 29% of them exhibiting cognitive impairment, a prevalence consistent with previous studies (Aarsland et al., 2021; Gonzalez-Latapi et al., 2021; Pfeiffer et al., 2014; Pigott et al., 2015). Additionally, the independent risk factors identified through logistic regression were incorporated into six machine-learning models for comparison. Logistic regression demonstrated superior predictive accuracy compared to other models, including Decision Tree, GNB, MLP, SVM, and KNN, making it the optimal method for classification modeling in this study. Furthermore, SHAP (Shapley Additive exPlanations) interpreters were employed to provide an intuitive explanation of how these variables predict the presence of cognitive impairment in PD patients. Finally, this study analyzed the correlation between cognitive assessment scores (MOCA and MMSE) and key gait-related motor parameters. The results indicated that PD patients with poorer cognitive scores exhibited worse gait-related parameters. These findings underscore the complex interplay between motor and cognitive dysfunction in PD patients and highlight a promising avenue for early, non-invasive diagnosis using wearable technology.

Parkinson’s disease (PD) is a multifaceted neurodegenerative disorder characterized by both motor and non-motor symptoms, including gait disturbances and cognitive impairment (Obeso et al., 2017). The relationship between gait parameters and cognitive impairment in PD patients has garnered increasing attention as a potential diagnostic tool for identifying cognitive decline in these individuals. This study highlights the identification of specific gait-related biomarkers that may serve as independent risk factors for cognitive impairment in PD patients, offering a non-invasive approach for monitoring disease progression and facilitating early intervention. Gait deficits in PD are characterized by reduced walking speed, primarily due to shorter step/stride length, as well as increased variability in each step (Hass et al., 2014; Mirelman et al., 2019; Morris et al., 1996; Morris et al., 2017). The biomarkers identified in this study include step length, walk speed, stride time, peak arm angular velocity, and peak angular velocity during steering. These findings are consistent with the existing literature, underscoring the role of gait abnormalities in cognitive decline among PD patients. For example, an earlier study by Camicioli et al. demonstrated that gait dysfunction in PD patients is associated with cognitive decline, with greater gait abnormalities correlating with poorer cognitive performance (Camicioli et al., 1998). Similarly, the study by Xie et al. indicated that gait disturbances in PD patients, particularly slower walking speed and reduced step length, are linked to cognitive impairment (Xie et al., 2019). A pioneering prospective cohort study by Fereshtehnejad et al. found that PD patients with cognitive impairment exhibited significant gait deficits, including reduced gait speed and increased stride variability (Fereshtehnejad et al., 2015). A large-scale longitudinal aging study conducted in the United Kingdom similarly identified slow walking speed as an independent risk factor for cognitive impairment (Hackett et al., 2018). Moreover, the study by Amboni et al. reported that changes in gait parameters, such as step length and walk speed, could predict cognitive decline in PD patients (Amboni et al., 2012). These findings are consistent with our results, which identified step length and walk speed as key predictors of cognitive impairment. The relationship between motor function and cognition in PD patients is complex; however, gait abnormalities are often early markers of cognitive decline, providing valuable insights into disease progression (Ho et al., 2020; Rasmussen et al., 2019; Stern et al., 2020). A recent study revealed that PD patients with cognitive impairment exhibit more pronounced gait disturbances compared to those without cognitive decline (Stern et al., 2020). Borges et al. found that PD patients with dementia had significantly slower walking speeds and shorter step lengths compared to those with PD alone (de Melo Borges et al., 2015). These findings align with our results, which showed that the greater the cognitive impairment, the poorer the gait performance, suggesting that gait analysis may serve as a reliable indicator of cognitive function in PD. Furthermore, the use of wearable sensors enables continuous gait monitoring, providing dynamic and real-time assessments of cognitive decline that can be integrated into clinical practice for ongoing patient management (Jayakody et al., 2022; Ma et al., 2019).

The integration of machine learning (ML) models encompasses a variety of algorithms, including Logistic Regression, Decision Tree, Gaussian naive Bayes (GNB), Multi-layer Perceptron (MLP), Support Vector Machine (SVM), and K-nearest neighbor (KNN), all of which are critical in our research. Logistic Regression emerged as the most accurate model for predicting cognitive impairment in PD patients, outperforming other machine-learning techniques. This finding aligns with previous studies, which indicate that Logistic Regression is often the preferred method for clinical prediction tasks due to its simplicity and interpretability (Christodoulou et al., 2019; Pavlou et al., 2016). Ariza et al. demonstrated that Logistic Regression when based on clinical and neuropsychological data, can effectively predict cognitive decline (Ariza et al., 2022). Similarly, Rijnen et al. found that Logistic Regression performs well in terms of interpretability and accuracy in clinical datasets, particularly when integrating clinical and biomechanical features (Rijnen et al., 2020). Although this study explored more complex models, such as SVM and MLP, their performance was inferior to logistic regression, potentially due to the relatively small dataset or the simplicity of the feature space. This underscores the importance of model selection based on dataset characteristics and clinical applicability (de Toro-Martín et al., 2018; Vergunst et al., 2019).

Our study also utilized the SHAP (Shapley Additive exPlanations) method to interpret the contribution of individual gait parameters to the model’s predictions. The application of SHAP analysis provides an interpretable framework to understand how each variable influences the model’s predictions of cognitive impairment. This is particularly critical in clinical settings, where transparent and interpretable models are essential for building trust and facilitating decision-making (Lundberg and Lee, 2017). As discussed by Xu and Lundberg et al., using SHAP to explain the contribution of each clinical parameter to disease prediction highlights the importance of interpretability in the clinical application of disease diagnostics (Lundberg et al., 2018; Xu et al., 2022). Similarly, Deng et al. emphasized the potential of SHAP and other interpretability tools to enhance the clinical applicability of machine learning models, enabling their use as decision-support tools in practice (Deng et al., 2022). Ghoraani and Costa et al. also employed machine learning algorithms to analyze gait data and predict cognitive decline, with similar findings indicating that gait parameters are key predictors of cognitive impairment (Costa et al., 2016; Ghoraani et al., 2021). The SHAP values in this study revealed that gait features such as step length and peak angular velocity during steering were the most important predictors of cognitive impairment, showing strong correlations with MOCA and MMSE scores. These findings provide valuable insights into the potential mechanisms underlying motor-cognitive interactions in PD. These findings are supported by previous studies that highlight gait speed as the “sixth vital sign” in aging populations, reflecting its sensitivity to both motor and cognitive decline (Adams et al., 2021; Fritz and Lusardi, 2009; Middleton et al., 2015). Moreover, the ability to visualize feature importance at the individual patient level offers a unique opportunity for personalized diagnosis, enabling clinicians to identify specific factors contributing to cognitive impairment in each case. By integrating clinical variables with SHAP, wearable sensor technology can be effectively utilized for the clinical diagnosis of PD.

An interesting aspect of our study analyzed the relationship between cognitive scales (MOCA and MMSE) and key motor parameters. The association between motor dysfunction and cognitive impairment may stem from shared neuropathophysiological mechanisms (Rosso et al., 2013; Rosso et al., 2017). Cognitive decline in PD patients is thought to result from the combined effects of dopaminergic depletion, cholinergic dysfunction, and structural alterations in cortical and subcortical regions (Delgado-Alvarado et al., 2016; Höglinger et al., 2004; Ray et al., 2018). On the other hand, motor dysfunction is linked to deficits in the basal ganglia, supplementary motor areas, and cerebellum, which are also implicated in cognitive abilities such as computation, attention, and executive function (Sigurdsson et al., 2022; Skidmore et al., 2022). The overlap between these neural circuits provides a plausible explanation for the observed correlations between motor parameters and cognitive scores. The correlations between MOCA/MMSE scores and motor parameters in this study further corroborate the link between motor and cognitive dysfunction in PD. Integrating motor analysis into clinical practice could enhance the accuracy of current cognitive screening tools, as MOCA/MMSE scores have been shown to have limitations in detecting early cognitive decline in PD (Marek et al., 2018). Combining motor parameters with traditional cognitive assessments allows clinicians to gain a more comprehensive understanding of patients’ cognitive status, facilitating personalized care plans addressing both motor and cognitive aspects of PD. Our study found that PD patients with lower cognitive scores also exhibited poorer motor parameter scores. Specifically, we observed that lower MOCA scores were significantly associated with slower walk speed, shorter step length, and abnormal turning velocity. These findings suggest that gait performance decline in PD patients parallels cognitive impairment. Walk speed showed a strong positive correlation with cognitive scores (MOCA: *R* = 0.640, *P* < 0.001; MMSE: *R* = 0.641, *P* < 0.001), consistent with Maidan et al.’s findings, suggesting that gait speed may serve as a reliable surrogate marker for cognitive function (Mone et al., 2022). The predictive value of this parameter may be particularly useful in clinical settings where comprehensive cognitive testing is not feasible. A study by Verghese et al. also reported that patients with cognitive impairment exhibited slower gait speed and shorter step length compared to cognitively intact individuals (Verghese et al., 2008). The significant correlation between MMSE scores and gait parameters, particularly step length and walk speed, further supports the notion that gait disturbances in PD patients may reflect underlying cognitive impairment. Step length also showed a positive correlation with cognitive scores (MOCA: *R* = 0.461, *P* < 0.001; MMSE: *R* = 0.643, *P* < 0.001). This finding aligns with Verghese et al.’s study, which demonstrated that shortened step length often precedes cognitive decline in PD patients (Morris et al., 2017). This relationship may reflect the shared neural basis between gait control and cognitive function, particularly within the frontal-cortical-subcortical circuitry. Although the correlation between MMSE scores and certain gait parameters (e.g., stride time) was not significant, this may be attributed to insufficient sample size or substantial inter-individual variability among patients. These non-significant findings suggest that relying solely on specific gait parameters may not comprehensively capture cognitive changes when analyzing cognitive impairment in PD patients. Additionally, Mielke et al.’s study indicated that gait speed could serve as an early warning signal for motor and cognitive decline in older adults (Mielke et al., 2013). Our analysis echoed this finding, as reduced gait speed was associated with lower cognitive scores. However, not all gait parameters are equally sensitive to cognitive decline. Lamoth et al.’s study found that certain gait parameters, such as gait variability, have stronger associations with cognitive impairment compared to others (Lamoth et al., 2011). These discrepancies may arise from differences in patient populations, study designs, or measurement tools, underscoring the need for more standardized approaches in gait analysis. Stride time was negatively correlated with MOCA scores (*R* = −0.174, *P* = 0.020), offering novel insights into the relationship between gait parameters and cognitive function. Although this correlation was not significant with MMSE scores (*R* = −0.078, *P* = 0.301), as noted by Allali et al., this trend highlights the complex relationship between temporal gait parameters and different aspects of cognitive function (Allali et al., 2017). Notably, the relationship between upper limb swing parameters and cognitive function, such as the weak positive correlation between peak arm angular velocities and cognitive scores (MOCA: *R* = 0.229, *P* = 0.002; MMSE: *R* = 0.195, *P* = 0.009), provides new directions for applying upper limb kinematics in cognitive assessments. This finding supports the study by Warmerdam et al., which suggested that increased arm swing variability may serve as an early indicator of motor and cognitive decline in PD patients (Warmerdam et al., 2021). The correlation between peak angular velocity during steering and cognitive scores (MOCA: *R* = 0.264, *P* < 0.001; MMSE: *R* = 0.296, *P* < 0.001) enhances our understanding of complex motor tasks in PD. This finding aligns with the results of Weiss et al., which demonstrated that turning difficulties often reflect both motor deterioration and higher-order cognitive processing deficits (Weiss et al., 2016). Collectively, these findings provide robust evidence for the intricate relationship between motor and cognitive domains in Parkinson’s disease. To assess whether the associations identified between motor parameters and cognitive scores were driven solely by between-group effects, we conducted additional correlation analyses within each MMSE-defined subgroup. The lack of statistical significance in these analyses may be attributable to reduced sample size and restricted score variability within subgroups, limiting the power to detect correlations. These findings suggest that the associations observed at the full-sample level are likely reflective of the continuous cognitive-motor interplay across the PD spectrum, rather than merely intergroup differences. Although BMI and frailty were not included in our current analysis, we recognize their potential relevance and plan to incorporate these measures in future studies using standardized assessment tools, such as the Clinical Frailty Scale and anthropometric indices, to better capture their impact on cognitive-motor interactions in PD.

In addition, we acknowledge that despite controlling for critical covariates including age, sex, disease duration, and LEDD, residual confounding may persist due to unmeasured factors such as sleep quality, mood status, and genetic predispositions. Additionally, given our hospital-based recruitment strategy, selection bias may exist, potentially inflating associations observed in our predictive models. While our machine learning approach enables high-dimensional pattern recognition and robust classification, it does not inherently resolve issues of model misspecification or unobserved confounders. As such, the results should be interpreted within the context of predictive modeling, not causal inference. Future studies with longitudinal designs, broader covariate inclusion, and causal inference techniques (e.g., instrumental variables) are warranted to further clarify underlying mechanisms. Although partial correlation analysis was not applied in the present study due to its primary focus on predictive modeling, this technique represents a valuable tool for future research exploring the interrelationship between gait and cognitive parameters in Parkinson’s disease, particularly within longitudinal or causal frameworks. Another significant limitation of this study is the exclusive use of brief screening tools (MMSE and MoCA) for cognitive evaluation. While practical and widely used, these tools lack the depth and specificity required to delineate domain-level cognitive deficits, and are not adequate for biomarker validation. According to Movement Disorder Society guidance, comprehensive Level II neuropsychological testing is recommended for research aimed at identifying biomarkers of cognitive impairment. Consequently, our reliance on screening scores constrains the diagnostic precision and generalizability of our findings. While our findings suggest a statistically significant relationship between gait characteristics and screening-based cognitive performance, we caution against interpreting these associations as indicative of direct causality or diagnostic capability. The cross-sectional nature of the study, along with the limitations of the cognitive tools employed, precludes firm conclusions regarding mechanistic or predictive inferences. Future studies should incorporate both longitudinal cognitive tracking and neuroimaging or electrophysiological biomarkers to better understand the nature and directionality of motor-cognitive interactions in PD.

In summary, this study demonstrates the feasibility and utility of using gait parameters derived from wearable sensors combined with machine learning to predict cognitive impairment in Parkinson’s disease. By identifying key gait parameters associated with cognitive decline and comparing the performance of machine learning models, we developed a predictive model that could serve as a valuable tool for the early detection of cognitive impairment in PD. The integration of gait analysis with machine learning technologies offers an exciting avenue for improving the management and outcomes of PD patients. Future research should aim to validate these findings in larger cohorts and explore the integration of gait analysis with other diagnostic tools to create a comprehensive diagnostic model for PD and its cognitive complications.

While this study provides valuable insights, it must again be emphasized that some limitations are acknowledged. First, although the sample size was sufficient for preliminary analyses, it may not fully represent the broader population of PD patients, particularly those at varying stages of the disease. Further studies with larger cohorts are required to validate these findings and assess their generalizability. Second, while we focused on gait parameters as predictors of cognitive impairment, other factors, such as executive function, sleep disturbances, and neuroimaging biomarkers, may also play critical roles in predicting cognitive decline in PD. Future research should incorporate a broader range of clinical and neuropsychological variables to enhance the predictive accuracy of the model. Furthermore, while wearable sensors provide valuable real-world data, their accuracy and reliability may vary depending on factors such as sensor placement, patient compliance, and environmental conditions. Advances in sensor technology and data processing algorithms are essential for improving the precision of gait analysis in clinical practice. Meanwhile, we acknowledge that our study does not distinguish mild cognitive impairment (MCI) from normal cognition. This limitation stems from the lack of neuropsychological test batteries and our focus on clinically significant cognitive decline. Future studies are warranted to incorporate MCI classification to enhance the granularity and early diagnostic value of gait-based biomarkers. Although the MMSE and MoCA are among the most commonly used tools for cognitive screening in PD, their utility remains limited in biomarker-oriented studies. These tools lack the sensitivity to detect subtle, domain-specific impairments and fall short of the standards recommended by the Movement Disorder Society. Future research will benefit from incorporating full neuropsychological batteries, domain-specific cognitive assessments, and longitudinal follow-up, which will enable a more refined understanding of how gait disturbances relate to distinct cognitive phenotypes and trajectories in Parkinson’s disease. Finally, cognitive status was assessed at a single time point, which restricts evaluation of progression and causality. Future longitudinal studies incorporating comprehensive neuropsychological testing are warranted.

## Data Availability

The original contributions presented in the study are included in the article/Supplementary material, further inquiries can be directed to the corresponding authors.

## Footnotes

1
https://cnsknowall.com
