# Supplementary material for: Modeling and validation of wearable sensor-based gait parameters in Parkinson’s disease patients with cognitive impairment
Source: Front Aging Neurosci. 2025 Jul 25;17:1590224. doi: 10.3389/fnagi.2025.1590224 (PMC12331692; doi:10.3389/fnagi.2025.1590224)
Supplement: Supplementary file 1 [file Data_Sheet_1.docx]

| Parameter | MoCA (Group A) | MMSE (Group A) | MoCA (Group B) | MMSE (Group B) |
| --- | --- | --- | --- | --- |
| Step Length | R=0.287, P=0.001 | R=0.349, P<0.001 | R=-0.015, P=0.917 | R=0.065, P=0.649 |
| Walk Speed | R=0.281, P=0.001 | R=0.285, P=0.001 | R=0.061, P=0.673 | R=0.018, P=0.903 |
| Stride Time | R=-0.024, P=0.794 | R=0.159, P=0.076 | R=-0.028, P=0.848 | R=0.186, P=0.192 |
| Peak arm angular velocity | R=-0.019, P=0.830 | R=-0.110, P=0.219 | R=-0.053, P=0.711 | R=-0.041, P=0.773 |
| Peak angular velocity during steering | R=0.213, P=0.016 | R=0.235, P=0.008 | R=0.048, P=0.736 | R=-0.045, P=0.756 |

Group A: PD patients without cognitive impairment.

Group B: PD patients with cognitive impairment.

Table S1. Subgroup Correlation Analyses


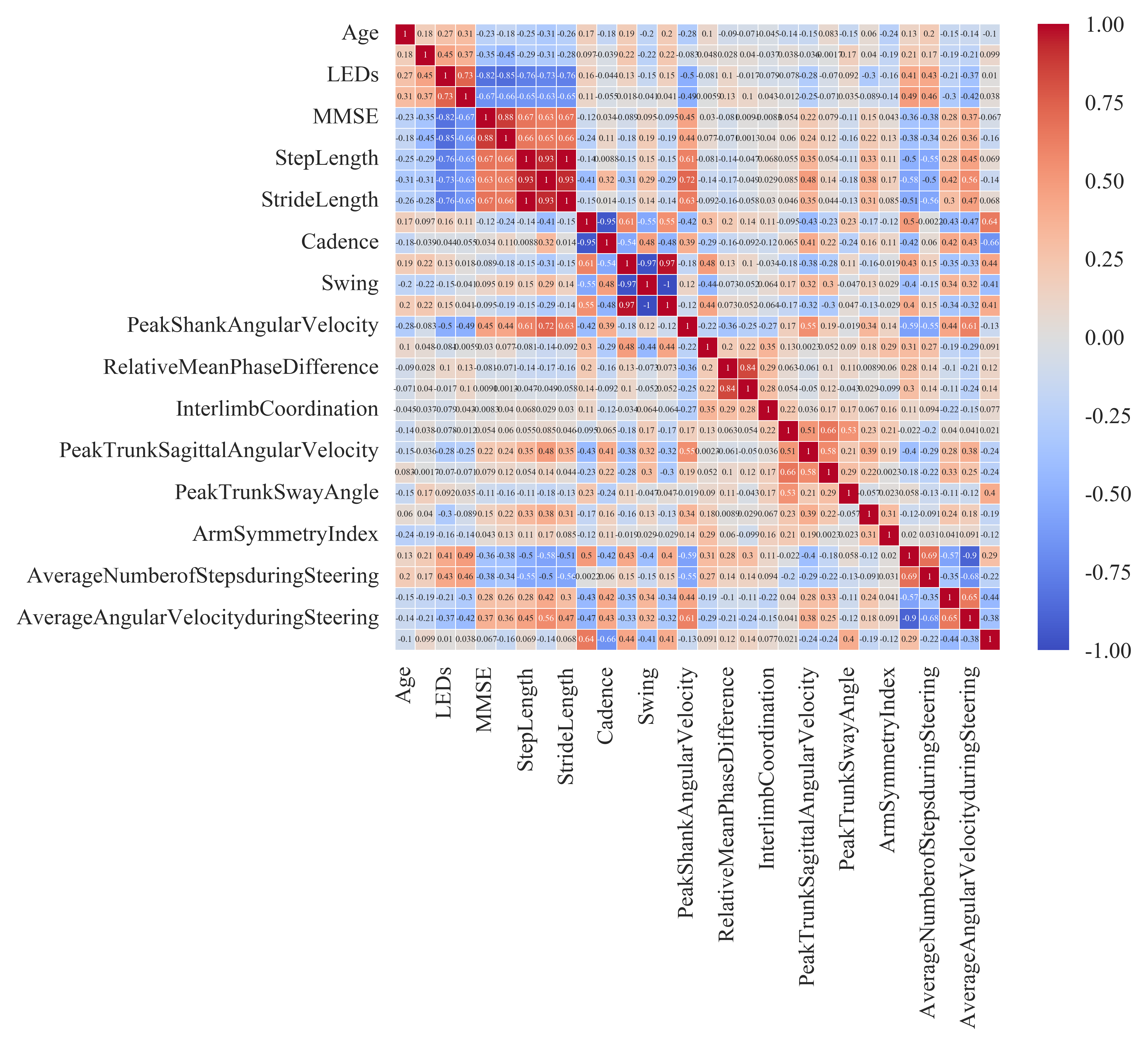


Supplementary Figure S1. Pearson correlation heatmap for all continuous independent variables used in the final model. Red indicates positive correlation, blue indicates negative correlation, and intensity reflects magnitude.


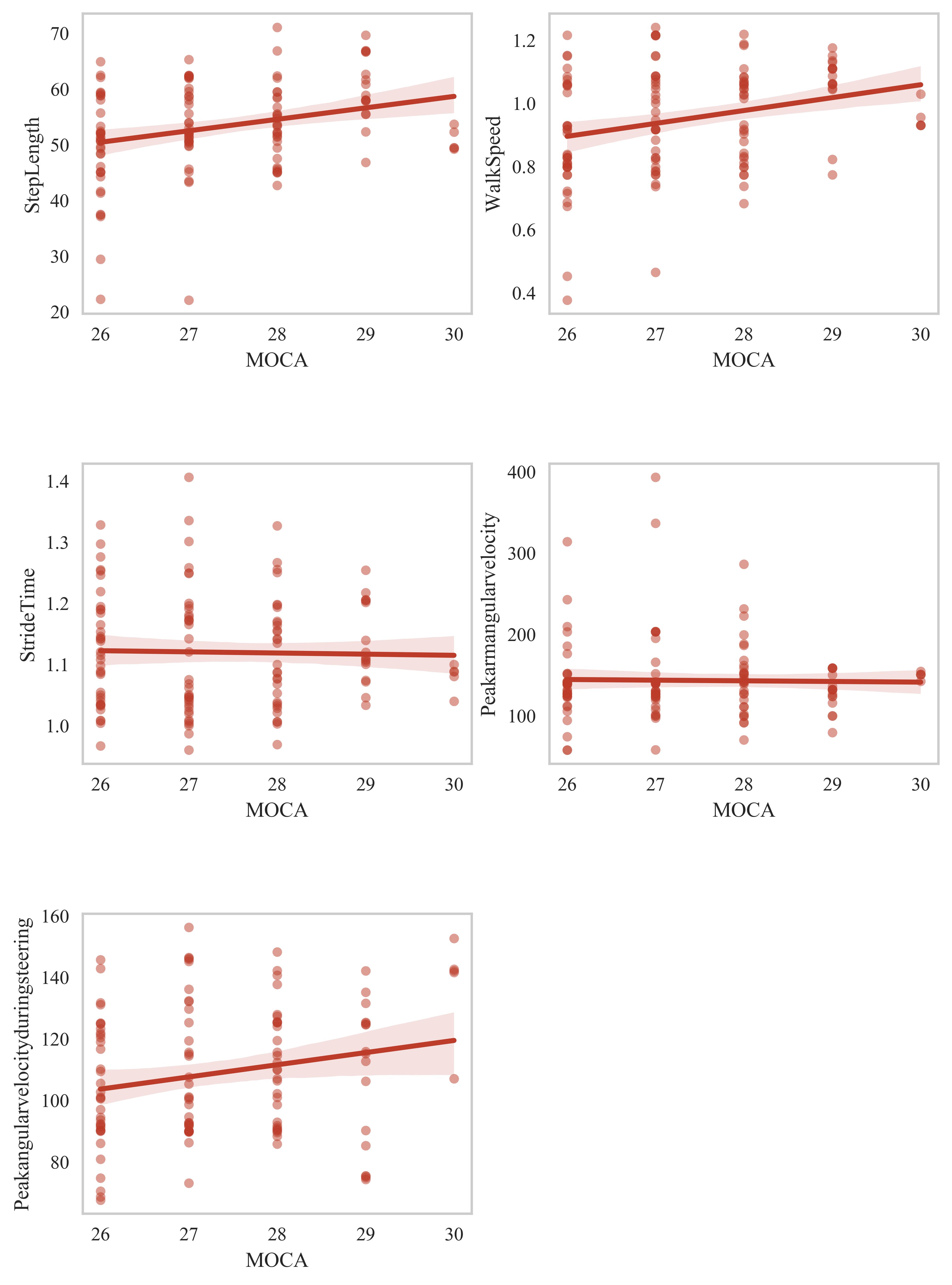


Figure S2. Correlation analysis of MOCA scores of Group A with step length, walk speed, stride time, peak arm angular velocity, and peak angular velocity during steering.


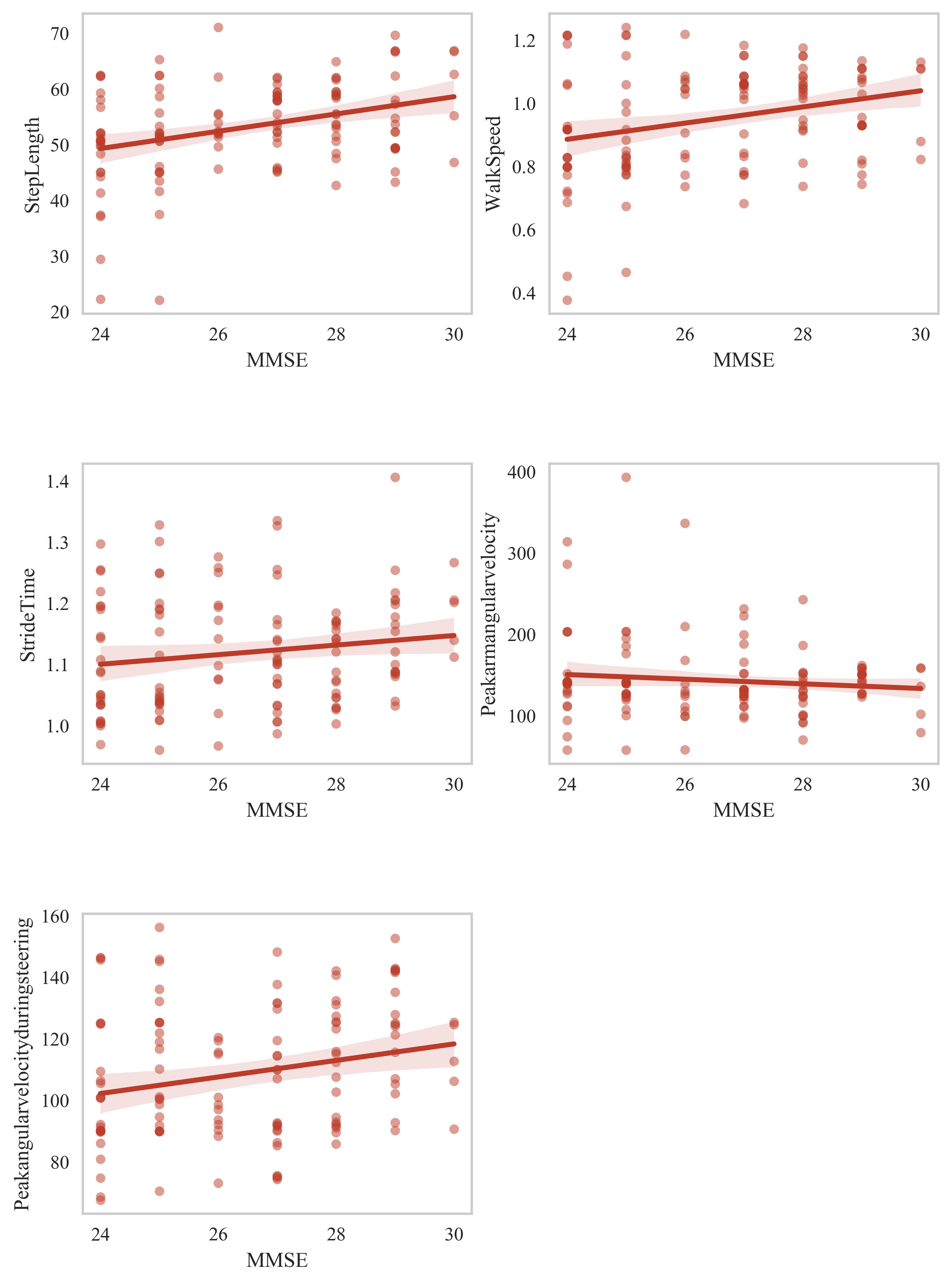


Figure S3. Correlation analysis of MMSE scores of Group A with step length, walk speed, stride time, peak arm angular velocity, and peak angular velocity during steering.


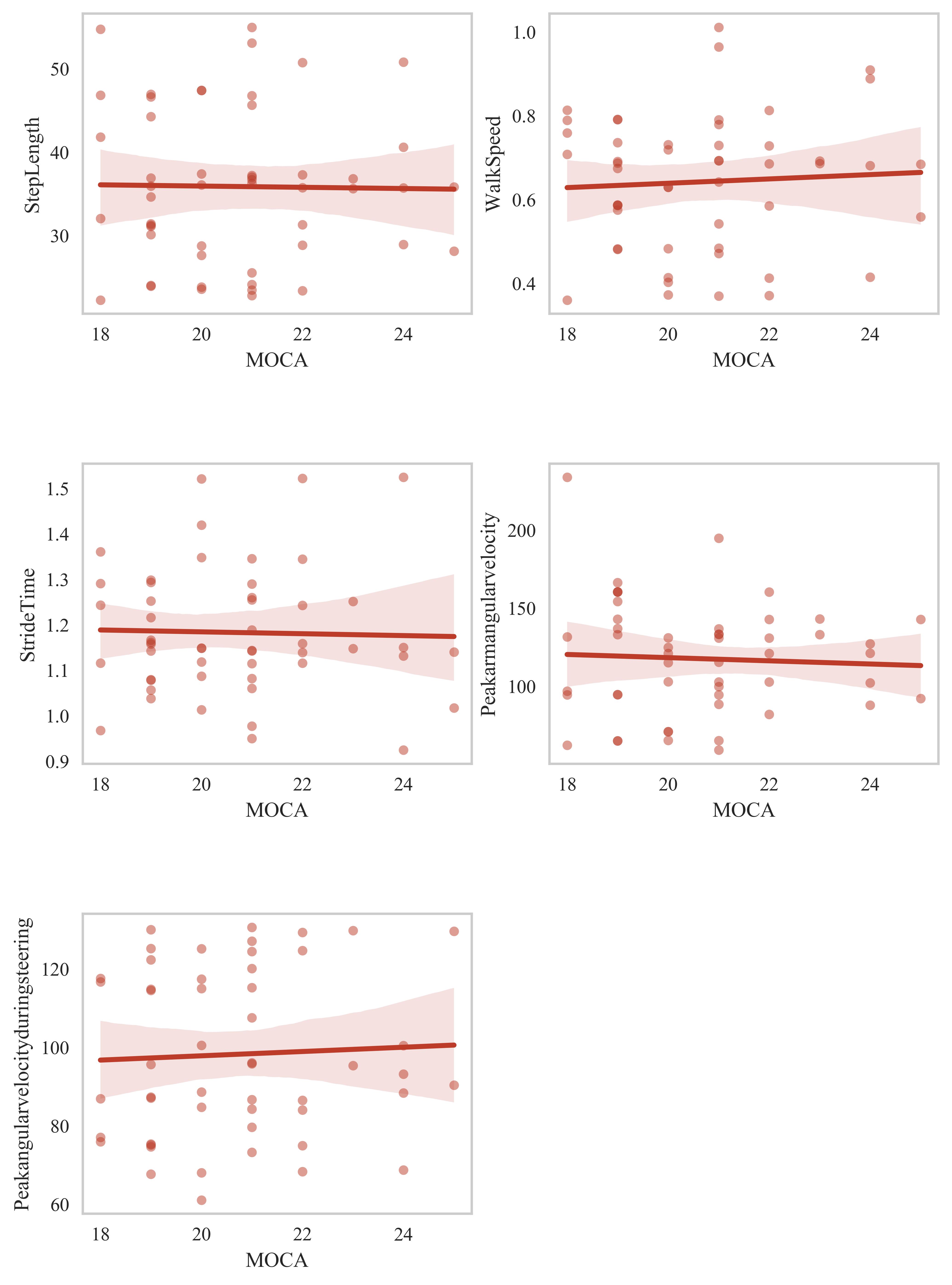


Figure S4. Correlation analysis of MOCA scores of Group B with step length, walk speed, stride time, peak arm angular velocity, and peak angular velocity during steering.


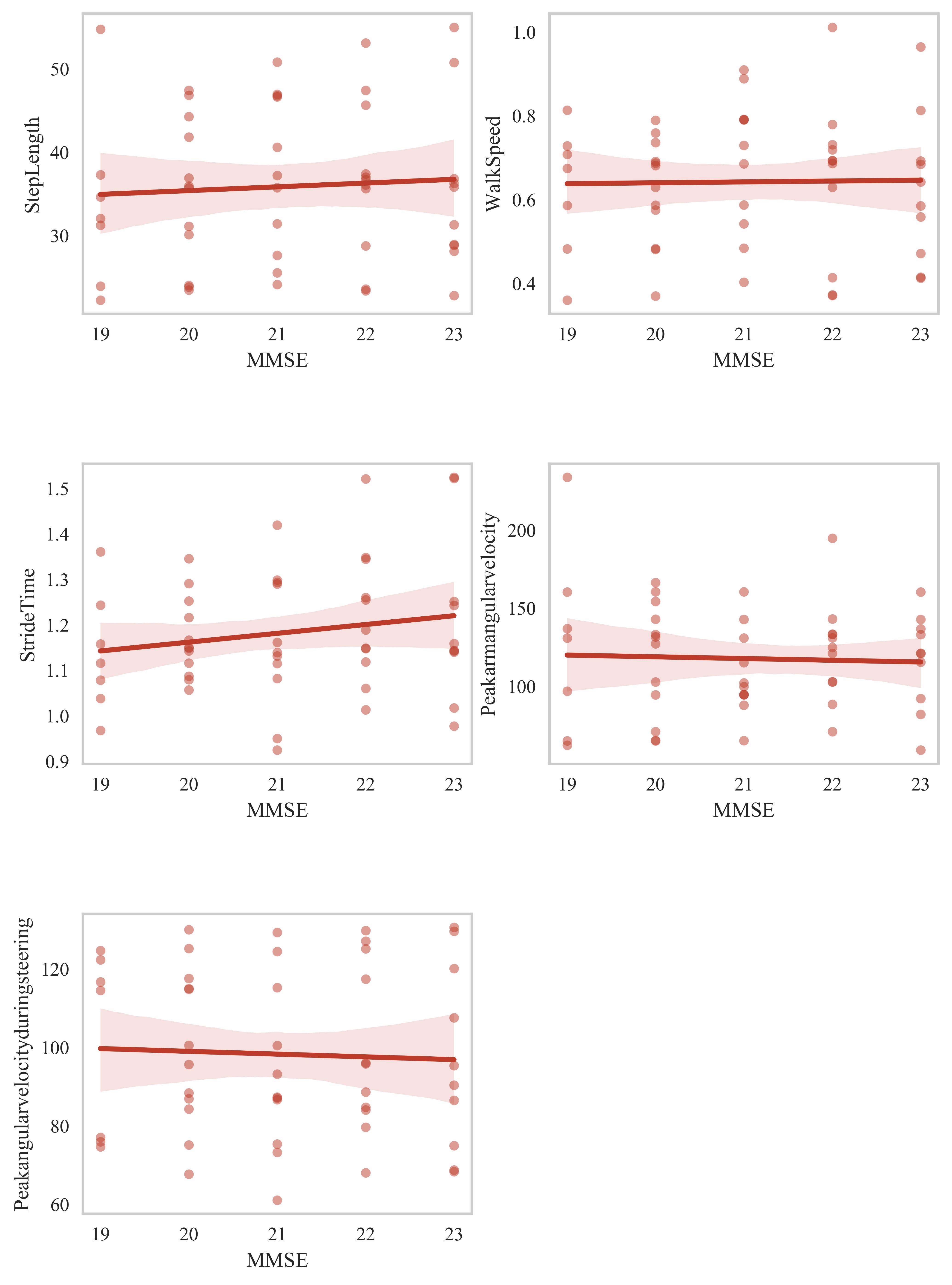


Figure S5. Correlation analysis of MMSE scores of Group B with step length, walk speed, stride time, peak arm angular velocity, and peak angular velocity during steering.
